# Crystal structure of 1-(4-formyl­benzyl­idene)-4-methyl­thio­semicarbazone

**DOI:** 10.1107/S1600536814016407

**Published:** 2014-08-01

**Authors:** Arantxa Pino-Cuevas, Rosa Carballo, Ezequiel M. Vázquez-López

**Affiliations:** a Departamento de Química Inorgánica, Facultade de Química, Edificio de Ciencias Experimentais, Universidade de Vigo, E-36310 Vigo, Galicia, Spain

**Keywords:** crystal structure, thio­semicarbazone, thio­urea, hydrogen bonding

## Abstract

The structure of the title compound, C_10_H_11_N_3_OS, comprises an approximately planar mol­ecule, with the r.m.s. deviation for the 15 non-H atoms being 0.089 Å. The conformation about the imine bond is *E* and an intra­molecular N—H⋯N hydrogen bond is evident. Mol­ecules are linked into a supra­molecular chain along the *b* axis by N—H⋯S hydrogen bonds.

## Related literature   

For the synthesis of the title compound, see: Jagst *et al.* (2005 ▶). For biological properties, see: Serda *et al.* (2012 ▶). For supra­molecular studies of thio­semicarbazones, see: Alonso *et al.* (2002 ▶).
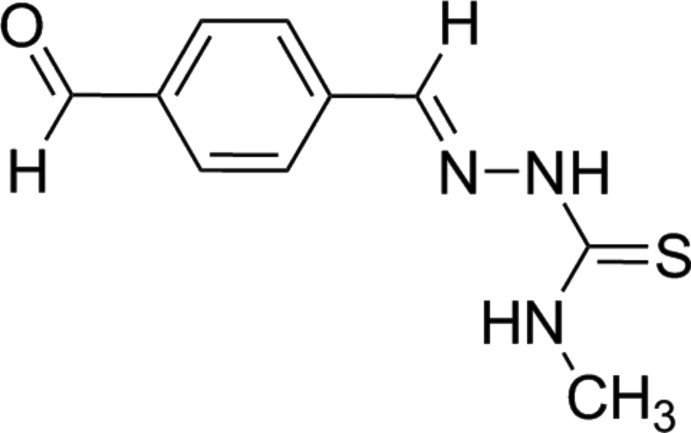


## Experimental   

### Crystal data  

C_10_H_11_N_3_OS
*M* *_r_* = 221.28
Orthorhombic, 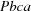

*a* = 13.1231 (3) Å
*b* = 8.8559 (2) Å
*c* = 19.3702 (4) Å
*V* = 2251.14 (9) Å^3^
*Z* = 8
Cu *K*α radiation
μ = 2.38 mm^−1^
*T* = 296 K
0.14 × 0.13 × 0.05 mm

### Data collection  

Bruker CCD SMART 6000 diffractometer
Absorption correction: multi-scan (*SADABS*; Bruker, 2007 ▶) *T*_min_ = 0.730, *T*_max_ = 0.898
22698 measured reflections
1986 independent reflections
1798 reflections with *I* > 2σ(*I*)
*R*_int_ = 0.046

### Refinement  

*R*[*F* ^2^ > 2σ(*F* ^2^)] = 0.034
*wR*(*F* ^2^) = 0.100
*S* = 1.08
1986 reflections
145 parameters
H atoms treated by a mixture of independent and constrained refinement
Δρ_max_ = 0.21 e Å^−3^
Δρ_min_ = −0.16 e Å^−3^

### 

Data collection: *APEX2* (Bruker, 2007 ▶); cell refinement: *SAINT* (Bruker, 2007 ▶); data reduction: *SAINT*; program(s) used to solve structure: *SHELXS97* (Sheldrick, 2008 ▶); program(s) used to refine structure: *SHELXL97* (Sheldrick, 2008 ▶); molecular graphics: *Mercury* (Bruno *et al.*, 2002 ▶); software used to prepare material for publication: *publCIF* (Westrip, 2010 ▶).

## Supplementary Material

Crystal structure: contains datablock(s) I, New_Global_Publ_Block. DOI: 10.1107/S1600536814016407/tk5328sup1.cif

Structure factors: contains datablock(s) I. DOI: 10.1107/S1600536814016407/tk5328Isup2.hkl

Supporting information file. DOI: 10.1107/S1600536814016407/tk5328Isup3.cml

**Fig. 1.**
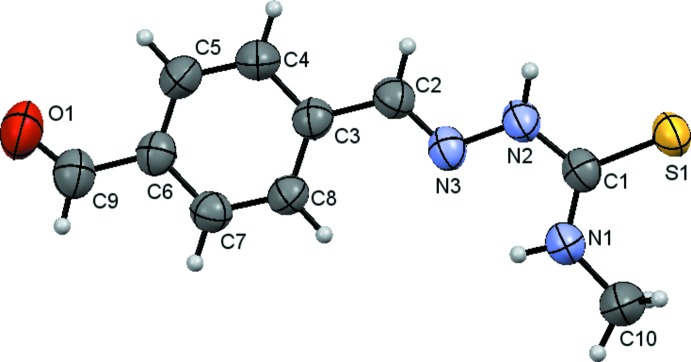
The molecular structure of the title compound showing the atom-labelling scheme and displacement ellipsoids at the 50% probability level.

. DOI: 10.1107/S1600536814016407/tk5328fig1.tif
The mol­ecular structure of the title compound showing the atom-labelling scheme and displacement ellipsoids at the 50% probability level.

**Fig. 2.**
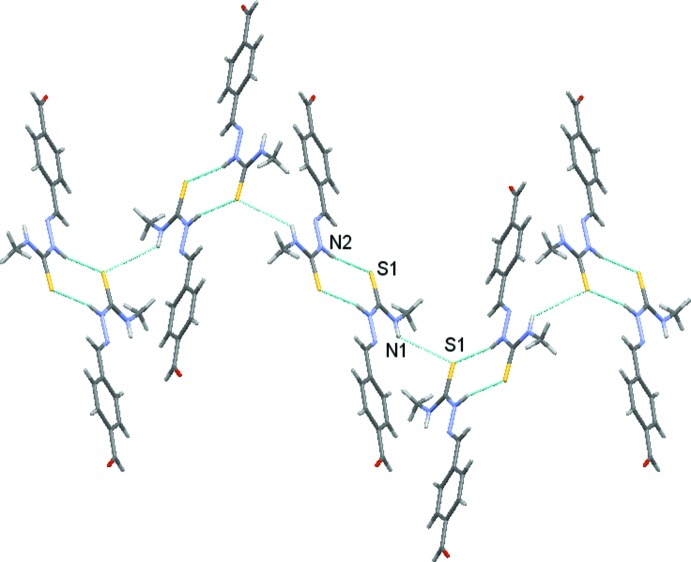
View of supramolecular chain formed by N—H···S interactions (dashed lines).

. DOI: 10.1107/S1600536814016407/tk5328fig2.tif
View of supra­molecular chain formed by N—H⋯S inter­actions (dashed lines).

CCDC reference: 1014062

Additional supporting information:  crystallographic information; 3D view; checkCIF report

## Figures and Tables

**Table 1:** Hydrogen-bond geometry (Å, °)

*D*—H⋯*A*	*D*—H	H⋯*A*	*D*⋯*A*	*D*—H⋯*A*
N2—H2N⋯S1^i^	0.902 (19)	2.53 (2)	3.4154 (14)	165.6 (16)
N1—H1⋯N3	0.84 (2)	2.238 (18)	2.6467 (18)	109.9 (15)
N1—H1⋯S1^ii^	0.84 (2)	2.992 (19)	3.5401 (15)	124.6 (16)

Symmetry codes: (i) 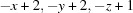; (ii) 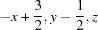.

## supplementary crystallographic information

### S3. Synthesis and crystallization

A solution of 4-methyl-3-thio­semicarbazide (392 mg, 3.72 mmol) in water (50 mL) was slowly added at 50°C to a solution of terephthaldicarboxaldehyde (500 mg, 3.73 mmol) in 100 mL water. Then the mixture was stirred at 50°C for 30 mins. Once cooled to room temperature, the yellow solid was filtered off and vacuum dried. Yellow single crystals suitable for X-ray diffraction were obtained by recrystallization from EtOH/H_2_O (1:1). Yield: 91%, M. pt: 213-216 °C. IR data (KBr, cm^-1^): 3368m, 3150m ν(N—H); 2838w, 2742w ν(C—H aldehyde); 1692 s ν(C═O); 1545 s, 1257m ν(C═N), 833m, 777w ν(C═S). ^1^H NMR data (DMSO-d_6_, ppm): 11.72 (s, 1H, N(2)—H); 10.03 (s, 1H, C(1)—H); 8.69 (s, 1H, N(2)—H); 8.11 (s, 1H, C(8)—H); 8.04 (d, 2H, J = 8.1 Hz, C(3,7)-H); 7.94 (d, 2H, J = 8.1 Hz, C(4,6)-H); 3.04 (s, 3H, C(10)—H).

### S4. Refinement

Carbon-bound H-atoms were placed in calculated positions (C—H = 0.95–0.99 Å) and were included in the refinement in the riding model approximation, with *U*_iso_(H) = 1.2*U*_eq_(C). The N-bound H-atoms were located in a difference Fourier map but were refined with distance restraints N—H = 0.84 (1) and 0.90 (1) Å, and with *U*_iso_(H) = 1.2*U*_eq_(N).

### Crystal data

**Table:**

C_10_H_11_N_3_OS	*F*(000) = 928
*M**_r_* = 221.28	*D*_x_ = 1.306 Mg m^−^^3^
Orthorhombic, *P**b**c**a*	Cu *K*α radiation, λ = 1.54178 Å
Hall symbol: -P 2ac 2ab	Cell parameters from 9894 reflections
*a* = 13.1231 (3) Å	θ = 4.6–66.6°
*b* = 8.8559 (2) Å	µ = 2.38 mm^−^^1^
*c* = 19.3702 (4) Å	*T* = 296 K
*V* = 2251.14 (9) Å^3^	Plate, yellow
*Z* = 8	0.14 × 0.13 × 0.05 mm

### Data collection

**Table:**

Bruker CCD SMART 6000 diffractometer	1986 independent reflections
Radiation source: fine-focus sealed tube	1798 reflections with *I* > 2σ(*I*)
Graphite monochromator	*R*_int_ = 0.046
φ and ω scans	θ_max_ = 66.6°, θ_min_ = 4.6°
Absorption correction: multi-scan (*SADABS*; Bruker, 2007)	*h* = −15→15
*T*_min_ = 0.730, *T*_max_ = 0.898	*k* = −10→10
22698 measured reflections	*l* = −22→22

### Refinement

**Table:**

Refinement on *F*^2^	Primary atom site location: structure-invariant direct methods
Least-squares matrix: full	Secondary atom site location: difference Fourier map
*R*[*F*^2^ > 2σ(*F*^2^)] = 0.034	Hydrogen site location: inferred from neighbouring sites
*wR*(*F*^2^) = 0.100	H atoms treated by a mixture of independent and constrained refinement
*S* = 1.08	*w* = 1/[σ^2^(*F*_o_^2^) + (0.0559*P*)^2^ + 0.3598*P*] where *P* = (*F*_o_^2^ + 2*F*_c_^2^)/3
1986 reflections	(Δ/σ)_max_ < 0.001
145 parameters	Δρ_max_ = 0.21 e Å^−^^3^
0 restraints	Δρ_min_ = −0.16 e Å^−^^3^

### Special details

**Table:**

Geometry. All e.s.d.'s (except the e.s.d. in the dihedral angle between two l.s. planes) are estimated using the full covariance matrix. The cell e.s.d.'s are taken into account individually in the estimation of e.s.d.'s in distances, angles and torsion angles; correlations between e.s.d.'s in cell parameters are only used when they are defined by crystal symmetry. An approximate (isotropic) treatment of cell e.s.d.'s is used for estimating e.s.d.'s involving l.s. planes.
Refinement. Refinement of *F*^2^ against ALL reflections. The weighted *R*-factor *wR* and goodness of fit *S* are based on *F*^2^, conventional *R*-factors *R* are based on *F*, with *F* set to zero for negative *F*^2^. The threshold expression of *F*^2^ > σ(*F*^2^) is used only for calculating *R*-factors(gt) *etc*. and is not relevant to the choice of reflections for refinement. *R*-factors based on *F*^2^ are statistically about twice as large as those based on *F*, and *R*- factors based on ALL data will be even larger.

### Fractional atomic coordinates and isotropic or equivalent isotropic displacement parameters (Å^2^)

**Table:**

	*x*	*y*	*z*	*U*_iso_*/*U*_eq_	
S1	0.90393 (3)	1.04683 (4)	0.40705 (2)	0.06222 (18)	
O1	0.88220 (12)	−0.01792 (18)	0.72752 (8)	0.0921 (5)	
N1	0.81376 (12)	0.78044 (16)	0.39148 (7)	0.0655 (4)	
C1	0.87314 (11)	0.86850 (16)	0.42864 (8)	0.0517 (3)	
N2	0.90945 (10)	0.81086 (15)	0.48868 (7)	0.0579 (3)	
C2	0.92328 (12)	0.62083 (19)	0.56504 (9)	0.0605 (4)	
H2	0.9617	0.6879	0.5913	0.073*	
N3	0.88781 (9)	0.66473 (14)	0.50704 (7)	0.0542 (3)	
C3	0.90556 (11)	0.46928 (19)	0.59137 (8)	0.0536 (4)	
C4	0.93836 (15)	0.43343 (19)	0.65777 (9)	0.0678 (4)	
H4	0.9707	0.5066	0.6844	0.081*	
C5	0.92354 (14)	0.2913 (2)	0.68448 (9)	0.0675 (4)	
H5	0.9454	0.2692	0.7290	0.081*	
C6	0.87602 (11)	0.18070 (18)	0.64526 (8)	0.0554 (4)	
C7	0.84324 (11)	0.21631 (18)	0.57902 (8)	0.0558 (4)	
H7	0.8112	0.1429	0.5524	0.067*	
C8	0.85745 (11)	0.35836 (17)	0.55224 (8)	0.0543 (4)	
H8	0.8349	0.3805	0.5079	0.065*	
C9	0.86069 (14)	0.0265 (2)	0.67155 (10)	0.0678 (4)	
H9	0.8313	−0.0428	0.6415	0.081*	
C10	0.76953 (19)	0.8229 (2)	0.32581 (10)	0.0924 (7)	
H10A	0.7165	0.8957	0.3332	0.139*	
H10B	0.7415	0.7351	0.3038	0.139*	
H10C	0.8213	0.8660	0.2968	0.139*	
H2N	0.9542 (15)	0.865 (2)	0.5138 (9)	0.075 (5)*	
H1	0.8010 (15)	0.695 (2)	0.4085 (9)	0.073 (6)*	

### Atomic displacement parameters (Å^2^)

**Table:**

	*U*^11^	*U*^22^	*U*^33^	*U*^12^	*U*^13^	*U*^23^
S1	0.0655 (3)	0.0429 (3)	0.0782 (3)	−0.00218 (15)	−0.01004 (18)	0.00628 (16)
O1	0.1022 (10)	0.0890 (10)	0.0851 (9)	0.0013 (8)	−0.0037 (8)	0.0320 (8)
N1	0.0797 (9)	0.0507 (8)	0.0662 (8)	−0.0120 (7)	−0.0135 (7)	0.0075 (6)
C1	0.0458 (7)	0.0469 (8)	0.0622 (8)	0.0028 (6)	0.0023 (6)	0.0008 (6)
N2	0.0550 (7)	0.0479 (7)	0.0707 (8)	−0.0065 (5)	−0.0097 (6)	0.0089 (6)
C2	0.0566 (8)	0.0555 (9)	0.0693 (9)	−0.0066 (7)	−0.0097 (7)	0.0047 (7)
N3	0.0477 (6)	0.0487 (7)	0.0663 (8)	−0.0014 (5)	−0.0001 (5)	0.0069 (6)
C3	0.0468 (8)	0.0555 (10)	0.0585 (9)	0.0000 (6)	−0.0032 (6)	0.0047 (6)
C4	0.0759 (11)	0.0641 (10)	0.0635 (9)	−0.0119 (8)	−0.0176 (8)	0.0023 (7)
C5	0.0763 (10)	0.0707 (11)	0.0555 (9)	−0.0044 (8)	−0.0131 (8)	0.0106 (8)
C6	0.0504 (8)	0.0573 (9)	0.0585 (8)	0.0032 (6)	0.0018 (6)	0.0059 (7)
C7	0.0539 (8)	0.0544 (9)	0.0590 (8)	−0.0013 (6)	−0.0037 (6)	−0.0017 (7)
C8	0.0536 (8)	0.0564 (9)	0.0531 (7)	0.0013 (6)	−0.0071 (6)	0.0040 (6)
C9	0.0656 (10)	0.0653 (10)	0.0724 (10)	0.0035 (8)	0.0001 (8)	0.0122 (8)
C10	0.1231 (18)	0.0784 (12)	0.0756 (11)	−0.0277 (12)	−0.0324 (12)	0.0131 (10)

### Geometric parameters (Å, º)

**Table:**

S1—C1	1.6829 (15)	C3—C8	1.392 (2)
O1—C9	1.187 (2)	C3—C4	1.393 (2)
N1—C1	1.317 (2)	C4—C5	1.375 (2)
N1—C10	1.448 (2)	C5—C6	1.388 (2)
C1—N2	1.356 (2)	C6—C7	1.390 (2)
N2—N3	1.3718 (18)	C6—C9	1.471 (2)
C2—N3	1.277 (2)	C7—C8	1.373 (2)
C2—C3	1.454 (2)		
			
C1—N1—C10	124.33 (15)	C4—C3—C2	118.99 (15)
N1—C1—N2	116.98 (14)	C5—C4—C3	120.82 (15)
N1—C1—S1	124.20 (12)	C4—C5—C6	120.26 (15)
N2—C1—S1	118.81 (12)	C5—C6—C7	118.98 (15)
C1—N2—N3	120.30 (13)	C5—C6—C9	121.80 (15)
N3—C2—C3	122.09 (15)	C7—C6—C9	119.21 (15)
C2—N3—N2	116.10 (13)	C8—C7—C6	120.97 (15)
C8—C3—C4	118.82 (15)	C7—C8—C3	120.15 (14)
C8—C3—C2	122.19 (14)	O1—C9—C6	126.24 (19)

### Hydrogen-bond geometry (Å, º)

**Table:**

*D*—H···*A*	*D*—H	H···*A*	*D*···*A*	*D*—H···*A*
N2—H2*N*···S1^i^	0.902 (19)	2.53 (2)	3.4154 (14)	165.6 (16)
N1—H1···N3	0.84 (2)	2.238 (18)	2.6467 (18)	109.9 (15)
N1—H1···S1^ii^	0.84 (2)	2.992 (19)	3.5401 (15)	124.6 (16)

Symmetry codes: (i) −*x*+2, −*y*+2, −*z*+1; (ii) −*x*+3/2, *y*−1/2, *z*.
