# Supplementary figures and images for: Crystal structure of 1-(4-formyl­benzyl­idene)-4-methyl­thio­semicarbazone
Source: Acta Crystallogr Sect E Struct Rep Online. 2014 Aug 1;70(Pt 9):o926. doi: 10.1107/S1600536814016407 (PMC4186068; doi:10.1107/S1600536814016407)

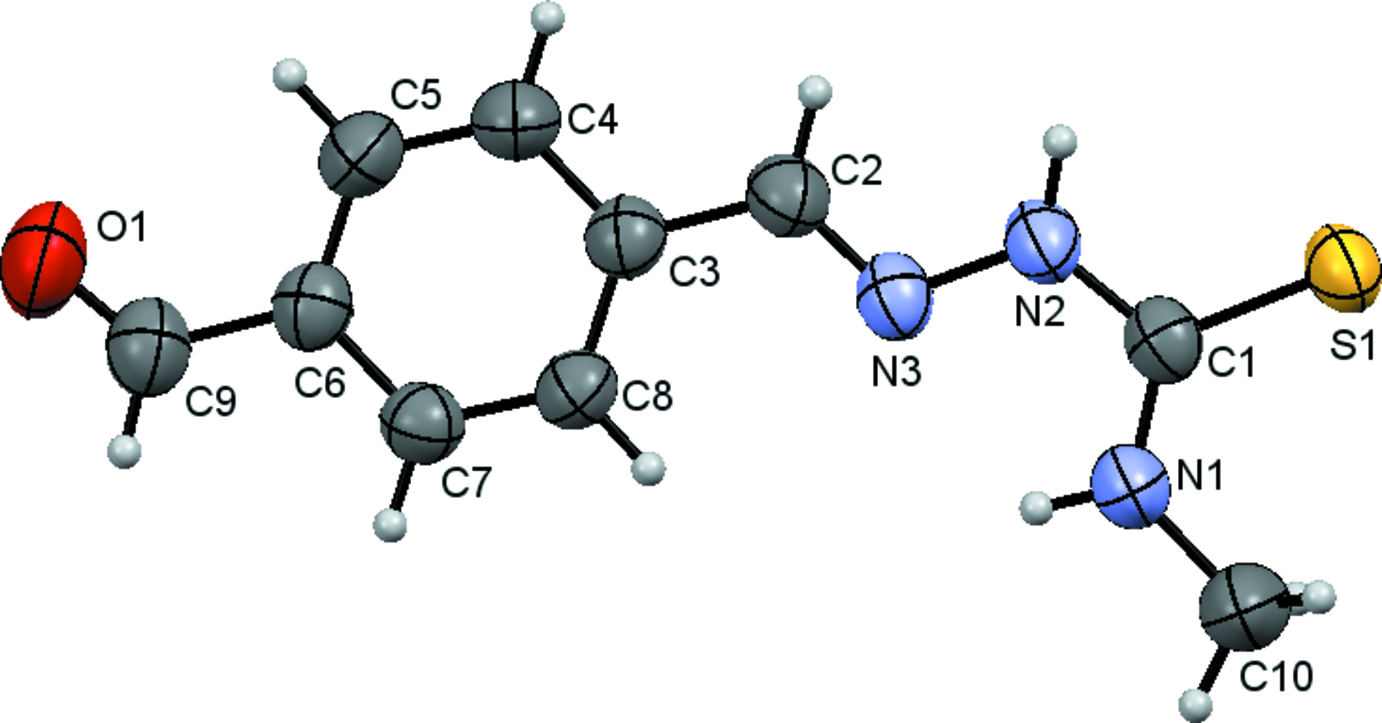

Supplement: Supplementary file 4 [file e-70-0o926-fig1.tif]

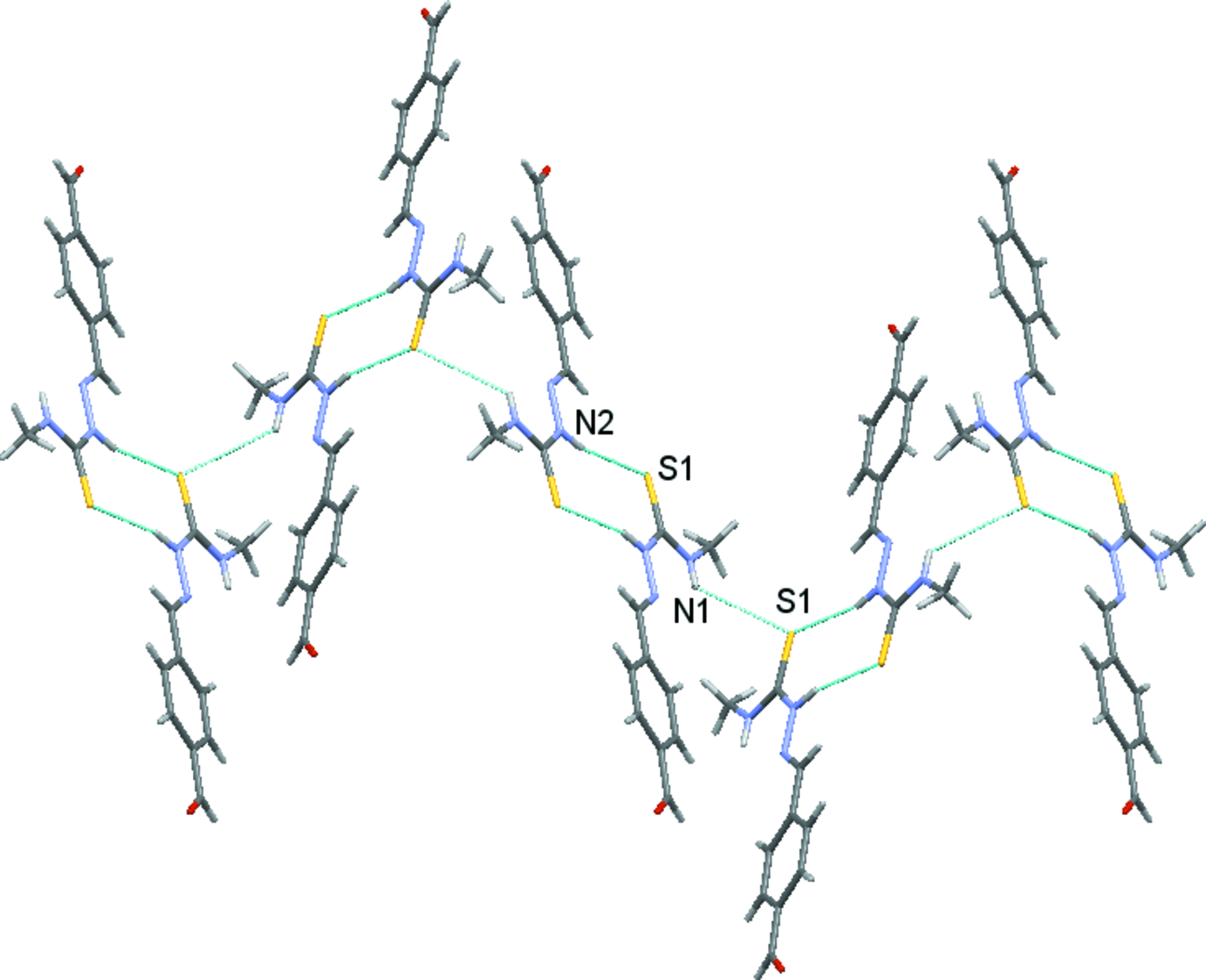

Supplement: Supplementary file 5 [file e-70-0o926-fig2.tif]
